# Effect of sonication and protease inhibitors on Elisa quantification of selected proteins in bovine udder tissue homogenates

**DOI:** 10.1038/s41598-026-38653-x

**Published:** 2026-02-05

**Authors:** Adrianna Szprynca, Michał Czopowicz, Magdalena Zalewska, Tomasz Sakowski, Jarosław Kaba, Emilia Bagnicka

**Affiliations:** 1 Department of Biotechnology and Nutrigenomics, Institute of Genetics and Animal Biotechnology, Polish Academy of Sciences, Postępu 36A, Jastrzębiec, 05-552 Poland; 2 Division of Veterinary Epidemiology and Economics, Institute of Veterinary Medicine, Warsaw University of Life Sciences-SGGW, Nowoursynowska 159c, Warsaw, 02-776 Poland; 3 Faculty of Biology, Institute of Microbiology, Department of Bacterial Physiology, University of Warsaw, Warsaw, 02-096 Poland

**Keywords:** ELISA, Sample preparation, Sonication, Protease inhibitors, Bovine mammary gland, Mastitis, Coagulase-positive staphylococci, Biochemistry, Biological techniques, Biotechnology, Microbiology

## Abstract

The protocols for preparing ELISA samples vary in terms of the use of mechanical homogenization (MH) alone or the additional use of protease inhibitors (PI) and/or sonication (S). It is unclear whether the type of protocol can affect the final protein yield; thus, we evaluated how different preparation methods and their combination affect the alpha-casein (CSN1), lactoferrin (LTF), and alkaline phosphatase (ALP) concentrations in udder parenchyma using a sandwich ELISA, and tested whether the udder health state (coagulase-positive staphylococcal ​​infection) affects the protocol efficacy. Samples from 22 cows (11 healthy, 11 infected) were processed using the four protocols. PI and S reduced protein concentrations, with the combination of MH + PI+S giving the lowest ​​values for all three proteins. Unlike CSN1, ALP was the most sensitive to processing. CSN1 levels were higher in infected samples, while lactoferrin and ALP were not influenced by infection. All preparation methods affected the protein levels, irrespective of health state. The highest protein concentrations were obtained using MH alone. Our findings highlight the need for careful optimization and standardization of sample preparation to ensure reliable and reproducible ELISA results.

**Supplementary Information:**

The online version contains supplementary material available at 10.1038/s41598-026-38653-x.

## Introduction

Immunoenzymatic tests can be used to quantify small amounts of specific substances with a high level of analytical specificity^1^. One of two widely-used and versatile enzyme-based immunotests is the Enzyme-Linked ImmunoSorbent Assay (ELISA), being a quantitative analytical method based on the antigen-antibody reaction^2^. Of all the varieties of ELISA used in modern laboratories, the most sensitive is the sandwich ELISA. The name refers to the multi-stepping procedure and the way that the protein is blocked between two antibody molecules^1^.

To obtain accurate results from ELISA, the samples must be prepared appropriately. Solid samples, such as tissues, must first be mechanically homogenized in an appropriate medium and diluted. While a range of diluents can be used for this purpose, such as phosphate-buffered saline (PBS), bovine serum albumin (BSA) or Tween, the choice of diluent should be based on the guidelines given in the protocol. PBS and BSA are often used to dilute serum and other biological samples, while Tween, as a non-ionic detergent, used in low concentrations, is good for preventing non-specific protein-protein interactions^3,4^.

A typical analysis, broadly speaking, consists of three stages: collecting the material, preparing the samples for testing, and carrying out the procedure itself. The latter is typically performed in accordance with a developed procedure or instructions included in the kit. In the case of commercial kits, the procedure and the reagents used may vary between producers. Such differences may be in the concentrations of some reagents outside the kit, the time or temperature of incubation, or even the method of preparing the samples for analysis.

While all protocols will require mechanical homogenization for solid material such as tissues, some protocols may also specify additional sonication or ultrasonic cell disruption during preparation. This added stage allows more thorough disruption of individual cells to release the desired content, as well as the remains of proteins and other undisrupted tissue, and to remove the any unnecessary components, such as DNA shearing^5^.

While it is crucial to release the desired protein from the cells, it is also necessary to prevent their degradation by various substances, such as proteases and phosphatases, released from the cells during processing, unless some degradation is intended to obtain the required effect or product. Otherwise, the presence of such components can be problematic in protein-focused analyses, and many kits intended for proteomic studies include protease and phosphatase inhibitors to prevent sample degradation. As protein degradation is generally highly undesirable, particularly in proteomic studies^6^, many protein extraction protocols include the use of protease inhibitors to prevent proteolysis, and the inhibitors are used in affinity chromatography for protein purification^7^. However, they should be handled with caution because they can interfere with the sample material during mass spectrometry^8^ by autofluorescing at the detection wavelength or signal drowning in the form of enzyme activity^9^.

Proteolytic enzymes such as proteases and peptidases enable the synthesis, activation and degradation of proteins by catalyzing the hydrolysis of peptide bonds. As such, in the cell, they must be strictly controlled to avoid excessive hydrolysis of the proteins needed for proper functioning. However, as they remain active in tissue samples collected after slaughter or biopsy^9^, proper sample storage and preparation is important under laboratory conditions. Samples containing protein or material intended for such analyses should be stored at low temperatures in clean solutions. High temperatures (37 °C and higher) and reactive environments such as those rich in reactive forms of oxygen, sugars, fatty acids or detergents should be avoided^11^.Maintaining a low temperature during sonication of tissue samples prior to ELISA is essential to limit the activation of endogenous proteases that can degrade target proteins. Cooling preserves protein integrity and antigenicity, ensuring accurate and reliable immunoassay results.

Alkaline phosphatase (ALP) occurs in nature, in many tissues and body fluids such as milk, where thanks to its high resistance to thermal inactivation, it is used to evaluate the pasteurization process. The presence of elevated ALP levels is mainly interpreted as insufficient pasteurization or contamination with raw milk^12^. Many milk products can be subjected to high temperatures because of the high thermal stability of the principal milk proteins, the caseins, which are synthesized and secreted in the mammary gland and are found nowhere else in nature. The main protein fraction of cow’s milk is constituted by casein alpha S1 (CSN1S1)^13^ while alpha-casein is defined as a major fraction of casein that consists of CSN1S1 and alpha-S2-casein (CSN1S2). These proteins are accompanied in milk by the whey proteins (β-lactoglobulin and α-lactalbumin), which are also synthesized in the mammary gland and by various others (e.g., bovine serum albumin and some immunoglobulins), which are derived from the blood. In turn, lactoferrin (LTF) is a protein produced by neutrophils that is found in milk and other body fluids. It has many biological functions related to the immune system, such as antimicrobial and anti-inflammatory activity, stimulation or inhibition of the antibody response and promotion of immune response cells. It also regulates the production and release of various cytokines, e.g. IL-1, IL-2, TNFα and IL-18^14^. As those mentioned proteins are always present in raw milk, they are suitable for evaluating the methods used to prepare secretory tissue for the ELISA test. Moreover, they were selected based on biological relevance to mammary gland function and host defense. α-Casein (CSN1) reflects secretory capacity and protein composition of milk, lactoferrin (LTF) is a key antimicrobial factor upregulated during mastitis and CoPS infection, and alkaline phosphatase (ALP) serves as an indicator of tissue metabolic activity and integrity. Differences in their extractability between tissue-preparation methods, while technically driven, may influence the interpretation of mammary immune status and functional responses in the context of infection. This highlights the importance of selecting appropriate workflows to preserve biologically meaningful proteins.

The mammary gland in dairy cattle is prone to infection by staphylococci, resulting in the development of mastitis. Some studies have found udder quarters infected with coagulase-positive staphylococci (CoPS) to have different gene expressions compared to healthy ones^15,16^. The presence of CoPS was used to indicate whether infection influences the efficiency of sample method preparation.

The aim of the study was to evaluate the influence of protease inhibition and sonication, alone or combined, and of the health state of the udder (i.e. healthy or infected with CoPS), on the protein concentration detected in bovine mammary glands. The analysis was performed using sandwich ELISA directed at the levels of three proteins, viz. casein alpha (CSN1), LTF and ALP, in the bovine udder parenchyma with secretory tissue predominance (ST).

## Results

**Fig. 1:**
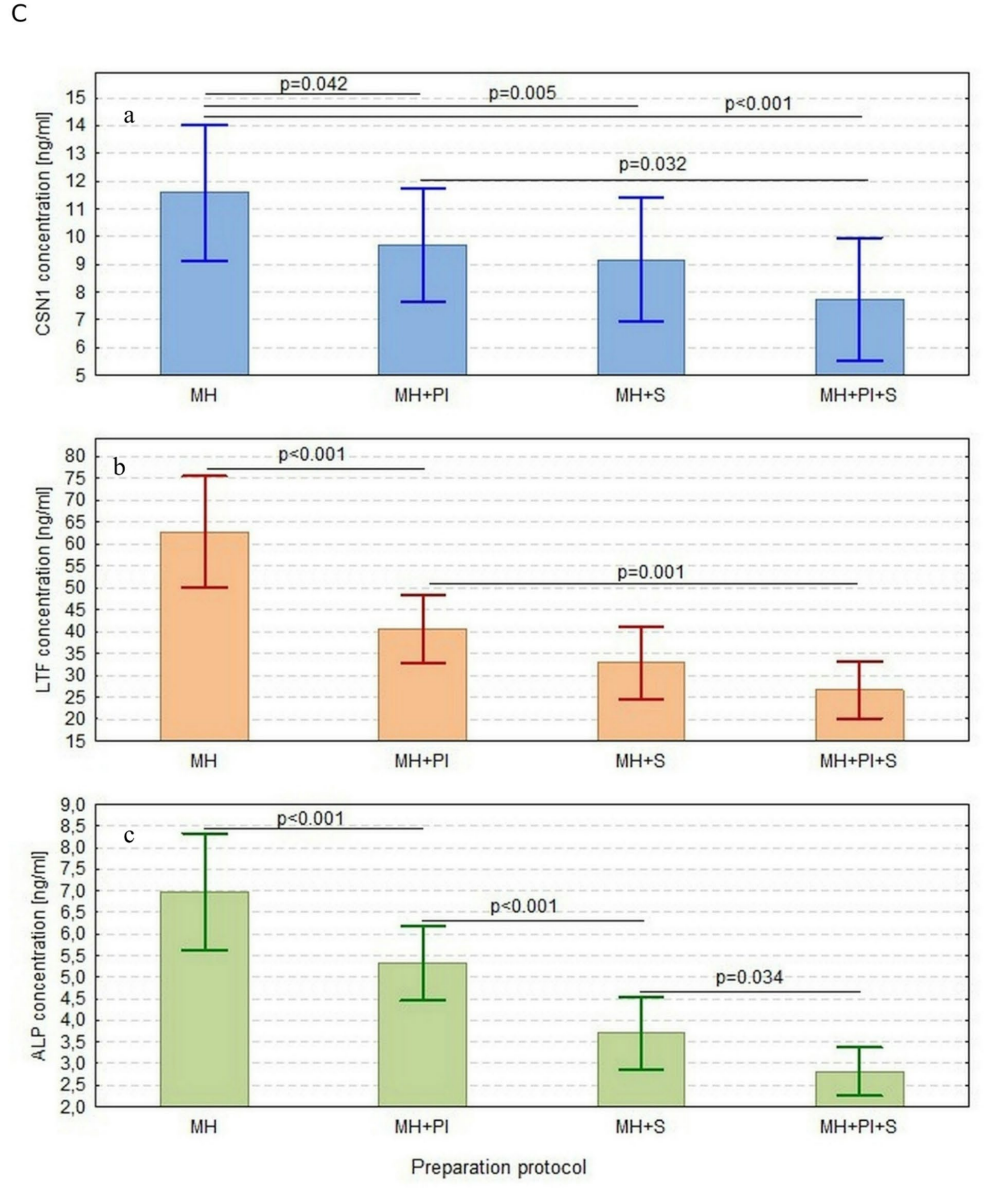
Concentration of: (**a**) casein alpha (CSN1), (**b**) lactoferrin (LTF), and (**c**) alkaline phosphatase (ALP) in the udder samples prepared using four different protocols: data presented as arithmetic means with 95% confidence intervals. Only significant differences indicated by the p-value. MH – Mechanical Homogenization, MH + PI – Mechanical Homogenization + Protease Inhibitors, MH + S – Mechanical Homogenization + Sonication, MH + PI+S – Mechanical Homogenization + Protease Inhibitors + Sonication

Regardless of the CoPS infection of the udder, the choice of sample preparation protocol had an influence on the concentration of CSN1 (F_3,60_=10.5, p *≤* 0.01) (Fig. 1a), LTF (F_3,60_=39.6, p *≤* 0.01) (Fig. 1b), and ALP (F_3,60_=68.1, p *≤* 0.01) (Fig. 1c) (Table S1).

Compared to the baseline MH protocol, the investigated protocols resulted in significantly lower yields of CSN1, LTF and ALP. In addition, lower CSN1 concentration between MH + PI+S than MH + PI (p *≤* 0.05) as well as lower LTF concentration in the same comparison (p *≤* 0.01) were noted, while no difference was observed between MH + PI and MH + S for CSN1 and LTF (*p* > 0.05) or between MH + S and MH + S+PI for the same proteins (*p* > 0.05) (Fig. 1a and b). ALP concentration was lower following MH + S than MH + PI (p0.01), and it was lower in MH + S+PI than in MH + S (p *≤* 0.05) or MH + PI (p *≤* 0.01) (Fig. 1c) (Table S1).

Only CSN1 concentration was affected by CoPS infection state - higher concentrations were noted in the CoPS-infected group (F_1,20_=8.92, p *≤* 0.01). The differences in LTF and ALP between healthy and infected groups were not significant (F_1,20_=3.24, *p* > 0.05 and F_1,20_=3.08, *p* > 0.05, respectively) (Fig. S1a, b, c). The relationships between sample preparation protocols, including infected groups, were insignificant for all proteins (CSN1: F_3,60_=0.11, *p* > 0.05; LTF: F_3,60_=1.07, *p* > 0.05; ALP: F_3,60_=2.41, *p* > 0.05), indicating that similar reductions in protein concentrations were noted between the CoPS-infected and healthy udders (Fig. S2a, b, c; Table S2).

**Fig. 2:**
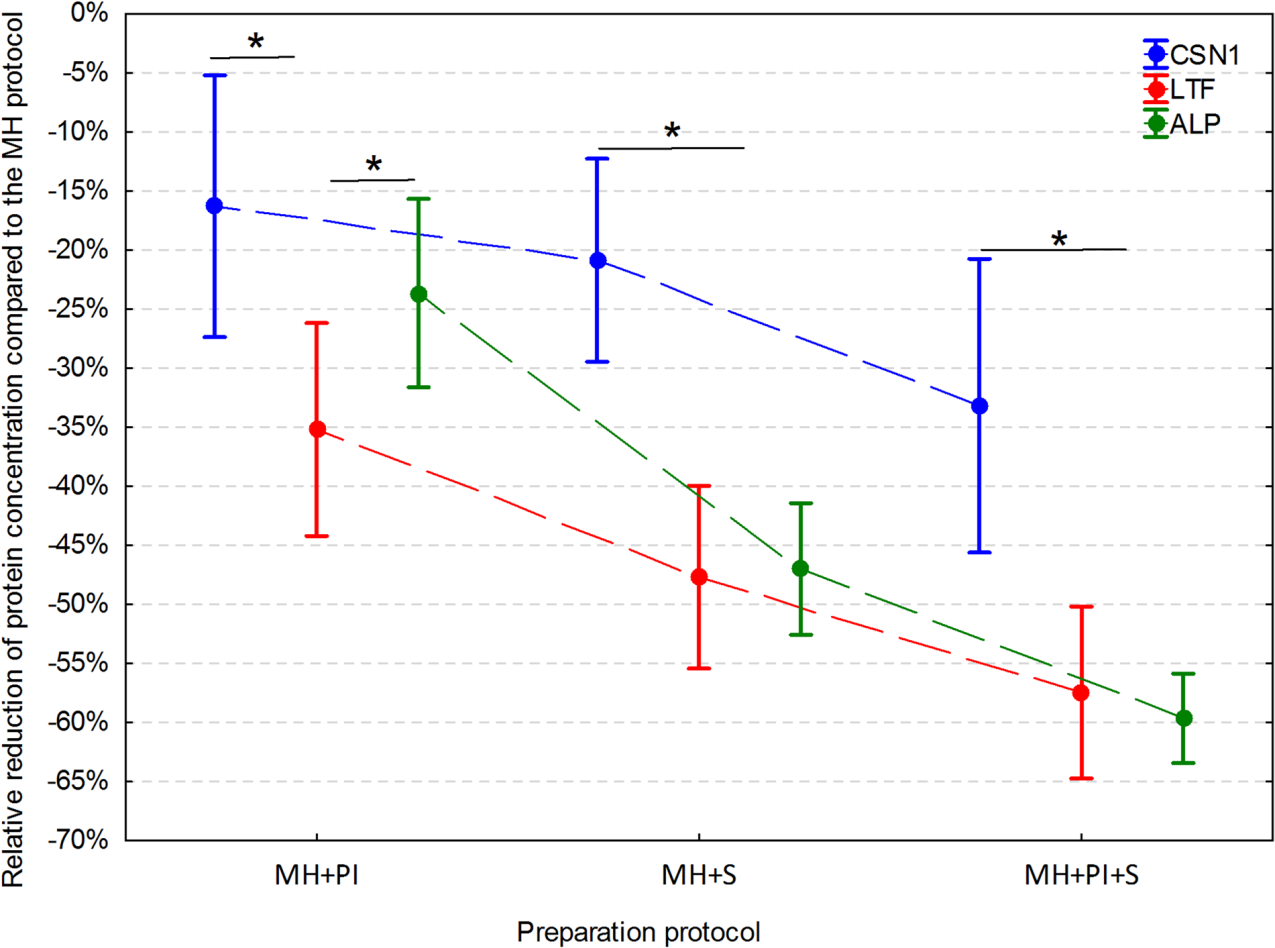
Mean relative reduction (95% confidence interval) of protein concentration with MH protocol as the baseline. MH – Mechanical Homogenization, MH + PI – Mechanical Homogenization + Protease Inhibitors, MH + S – Mechanical Homogenization + Sonication, MH + PI+S – Mechanical Homogenization + Protease Inhibitors + Sonication. Significant differences between proteins (α = 0.05) indicated by an asterisk (*).

When samples processed using the MH + PI protocol were compared to the baseline MH protocol, the mean CSN1 concentration was reduced by 1.9 ng/ml (CI 95%: 0.4, 3.4 ng/ml; relative reduction 16%; CI 95%: 5%, 27%), the mean LTF concentration by 22.1 ng/ml (CI 95%: 13.4, 30.7 ng/ml; relative reduction 35%; CI 95%: 26%, 44%), and the mean ALP concentration by 1.7 ng/ml (CI 95%: 0.91, 2.4 ng/ml; relative reduction 24%; CI 95%: 16%, 32%). In the MH + PI protocol, a significantly larger relative reduction of LTF concentration was noted compared to CSN1 and ALP (p *≤* 0.01) (Fig. 2).

When samples processed using the MH + S protocol were compared to the baseline MH protocol, the mean CSN1 concentration was reduced by 2.4 ng/ml (CI 95%: 1.3, 3.5 ng/ml) (relative reduction 21%; CI 95%: 12%, 29%), the mean LTF concentration by 29.9 ng/ml (CI 95%: 22.5, 37.3 ng/ml) (relative reduction 48%; CI 95%: 40%, 55%), and the mean ALP concentration by 36.0 ng/ml (CI 95%: 26.9, 45.1 ng/ml) (relative reduction 47%; CI 95%: 41%, 53%). In the MH + S protocol, a significantly larger relative reduction of LTF and ALP concentrations was noted compared to CSN1 (p *≤* 0.01) (Fig. 2).

When samples processed using the MH + PI+S protocol were compared to the baseline MH protocol, the mean CSN1 concentration was reduced by 3.8 ng/ml (CI 95%: 2.2, 5.5 ng/ml; relative reduction 33%; CI 95%: 21%, 46%), the mean LTF concentration by 36.0 ng/ml (CI 95%: 26.9, 45.1 ng/ml; relative reduction 57%; CI 95%: 50%, 65%), and the mean ALP concentration by 4.2 ng/ml (CI 95%: 3.3, 5.0 ng/ml; relative reduction 60%; CI 95%: 56%, 63%). In the MH + S+PI protocol, a significantly larger relative reduction of LTF and ALP concentrations was noted compared to CSN1 (p *≤* 0.01) (Fig. 2).

## Discussion

Our findings confirm that the protocol used for the preparation of mammary gland samples for ELISA testing affects the concentration of CSN1, LTF and ALP. Both protease inhibitors (PI) and sonication (S) reduce protein concentrations, and the combination of PI with S exerts the strongest effect. Similar effects were noted between udder quarters regardless of the health status. Although the study generated comparative ELISA results, several limitations should be considered. This manuscript focuses on the impact of tissue-preparation methods on ELISA outcomes, rather than on the underlying biochemical mechanisms. Total protein quantification was not performed, tissue material was limited, and no orthogonal validation of matrix effects (e.g., spike–recovery or Western blot) was included, limiting differentiation between assay interference, protein degradation, or epitope masking; thus, future studies more focused on mechanical background are required.

Various techniques are used to determine the presence of protein and its concentration, although the most common are Western Blot (WB), protein microarrays and ELISA tests. However, the latter allows a precise determination of the concentration of a specific protein in the material. In WB, sample preparation typically begins with the lysis of cells or tissues using a lysis buffer with protease/phosphatase inhibitors, with ultrasound sonication being a suitable homogenization method^17^. Microarray analysis protocols recommend using protease or phosphatase inhibitors where proteins need to be stored for a long period, or the laboratory analysis cannot be performed quickly^18^. Using inhibitors is even recommended by some manufacturers^19^.

Ultrasonic treatment, i.e. sonication, is a popular method of sample disruption as it combines cell lysis and non-specific DNA shearing. However, sonication can potentially degrade proteins. Pchelintsev et al.^20^ report that large proteins, similar in size to genomic DNA, are susceptible to degradation by ultrasonic treatment and recommend that the optimal sample volume for sonication should be 500 µl per 1.5 ml tube (ratio 1:3). This is close to the volume used in the present study, i.e. 700 µl in a 2 ml tube, being a ratio of 1:2.86. Like Pchelintsev et al.^20^, our present findings also indicate that sonication resulted in lower concentrations of the tested proteins compared to the control (mechanical homogenization).

In other studies of sonication, Özbek and Ülgen^21^ also observed a decrease in the activity of alcohol dehydrogenase (ADH), malate dehydrogenase (MDH), glucose-6-phosphate dehydrogenase (G6PDH), L-lactic dehydrogenase (LDH) and β-galactosidase (βG) with a rise in acoustic power. However, ALP level was found to be stable, and the acoustic power did not affect its activity. As such, they propose that sonication parameters should be individually adjusted to the enzyme being studied because both the time and intensity of sonication can lead to enzyme degradation while disrupting cells. However, in contrast, our present findings indicate that the ALP concentration was substantially lower when sonication was combined with mechanical homogenization.

Liu et al.^22^ found prolonged sonication to cause significant protein degradation, which they attribute to an increase in temperature. Mishra et al.^23^ propose that sonication is an effective tool for disrupting cells and partly homogenized tissues but it is not effective for solid tissues, also due to the heat generated during this method. Therefore, it is important to use short sonication cycles and cool the samples between them or keep them at a constant temperature (e.g. in water baths), as used in our procedure. However, in our case, breaking the sonication and cooling samples between sonication cycles did not prevent partial degradation of the protein. Nevertheless, strict adherence to the protocol and uniform treatment of samples resulted in similar degradation rates in all samples. Interestingly, the samples that underwent sonication demonstrated a lower range of each protein concentration compared to the samples that did not. This may mean that sonication, by releasing more proteins from the inside of cells, causes smaller differences between the CPS and H groups, although some of the proteins are degraded, as indicated by the difference between homogenization alone and sonicated samples.

The disruption of cell structures may involve the release of enzymes, including proteases. The latter must be inhibited effectively to determine the exact concentration of these proteins in a sample. Indeed, protease inhibitors are commonly used in protein analysis methods, such as WB. Depending on the manufacturer and type of material, protease inhibitors may be an essential part of sample preparation^24–27^, however, they are often omitted when preparing samples for ELISA tests as they interfere with the assay procedure or interact non-specifically with the tested protein or enzyme.

Zhang^9^ proposes that interference with signal detection may be caused by autofluorescence of the inhibitors at the detection wavelength or by signal drowning in the form of enzyme activity. The author also divides protease inhibitors into false inhibitors, where their influence appears to decrease substrate concentration, and activators, when an increase in product concentration is visible. The author also notes that inhibitors should be tested in advance, and if such irregularities are observed or detected, the chromatography protocol should be changed or substrates and products should be monitored.

Otherwise, no other problems have been reported with protease inhibitors unless they are used as therapies for diseases, e.g. AIDS^28^. In the present study, the use of a protease inhibitor cocktail when preparing samples for ELISA tests resulted in a decrease in the concentration of three studied proteins, including one enzyme. In addition, combining protease inhibitors with sonication resulted in a greater decrease in protein levels compared to samples subjected to mechanical homogenization alone.

Regarding the influence of the state of health of the mammary gland, only CSN1 differed between healthy and CoPS infected quarters. The method of preparation did not demonstrate any effect. The LTF and ALP concentrations tended to be higher in the CoPS group. However, when using the interaction between state of health and preparation method in the statistical model, no differences were found between subgroups, perhaps because of the high intragroup variation of the traits. Indeed, the coefficient of variation (CV) for CSN1 was the highest (48.63), compared to 45.78 for LTF and 42.12 for ALP, however, all of them were very high.

The finding that CSN1 concentration was higher in CoPS-infected quarters contradicts many previous studies, which show a decrease in casein contents, for example, Johansson et al.^29^ report reduced total casein content, including lowered CSN1S1, A1 and A2 β casein levels in UHT milk inoculated with *Staphylococcus aureus* strain, compared with control milk. They attribute this to the action of bacterial proteases. Zalewska et al.^30^ report that in subclinical mastitis, the concentrations of milk proteins remained stable despite the presence of bacterial infection, including CoPS such as *S. aureus*. Perhaps the differences are due to different types of mastitis, i.e. clinical vs. subclinical mastitis.

In our present study, it was expected that LTF and ALP protein concentrations would be higher in the CoPS groups. LTF plays an important role in mastitis as it has antimicrobial and anti-inflammatory activity, promotes the immune response and regulates the production and release of some cytokines^14^. Also, ALP can be considered as an early indicator of subclinical mastitis^31^.

## Conclusions

Our findings indicate that when preparing bovine udder samples, the use of sonication, protease inhibitors, or both combined, decreases the concentration of all analyzed proteins, and this effect occurs regardless of the health condition of the udder. Furthermore, sample preparation methods based on sonication (i.e. MH + S, MH + S+PI) have a less strongly effect on CSN1 than LTF or ALP. Interestingly, sonication reduces protein concentration more than the use of protease inhibitors, and the combination of sonication and protease inhibitors (MH + S+PI) results in greater reduction of ALP than the use of a single procedure (MH + S or MH + PI). Finally, despite the manufacturer’s recommendation that the samples should be sonicated, the highest protein concentrations were obtained using homogenization alone; however, further study is needed. While this study demonstrates that tissue-preparation methods significantly influence ELISA outcomes, further research is needed to refine and extend these findings. Future studies should incorporate total protein normalization and orthogonal analytical approaches to mechanistically distinguish between protein degradation, epitope masking, and differences in extractability. Additionally, systematic evaluation of processing parameters, such as homogenization intensity and temperature control, will be essential to optimize tissue-preparation workflows for ELISA-based analyses.

## Materials and methods

### Cows and Microbiological tests

The study was conducted on 50 Polish Holstein-Friesian dairy cows being between their first and fourth lactations. They were maintained at the Experimental Farm of the Institute of Genetics and Animal Biotechnology in Jastrzębiec, Central Poland. The details of animal housing are described by Kościuczuk et al.^16^ and the feeding conditions by Korwin-Kossakowska et al.^32^. Briefly, cows were fed exact TMR. The average milk yield around 9000 kg per lactation. The median somatic cell count (SCC) values were 1.3 × 10^6^ for the cows in the CoPS group. The cows with the whole healthy udder had a median SCC of 0.1 × 10^6^ during the whole of their last lactation. The animals used in the study had been slaughtered in a certified abattoir under constant veterinary supervision on approximately the 280th day of lactation (± 25). The reason for slaughter was chronic, subclinical mastitis, or for cows without udder infection, reproductive problems. All cows were slaughtered to obtain meat for commercial purposes. The animals with udder problems had been treated with several unsuccessful antibiotic therapies in their last lactation, but the treatment was completed at least one month before slaughter.

Two days before slaughter, foremilk samples were collected manually and aseptically into a sterile container (Medlab, Raszyn, Poland), separately from each udder quarter and tested microbiologically. The details of microbiological tests were described by Kościuczuk et al.^15^. Samples of the mammary gland parenchyma were taken from each udder quarter immediately after slaughter. All samples (1 cm × 1 cm × 5 cm) were taken from tissue containing a predominance of ST, deep in the upper part of the gland. The collected material was washed out in PBS, frozen and transported in liquid nitrogen, and then stored in −80 °C for further analyses. A total of 22 udder quarter samples were selected from 200 and divided into two groups based on the microbiological analysis results of the milk samples: (1) a CoPS group comprising eleven udder samples from cows infected with CoPS and (2) a control group (H) comprising eleven samples from cows with samples negative for CoPS from all udder quarters. Each sample came from a different cow. Some cows were infected with coagulase-negative staphylococci or had mixed infections, and such cows were excluded from the study. When neighboring quarters were infected with different pathogens, they were also excluded from the analyses. Moreover, although the number of CoPS samples was 27 and H = 13, we decided to use 11 samples per group to perform analyses on single ELISA plates.

ELISA tests and sample preparation protocols.

The concentrations of CSN1, LTF and ALP were determined using three sandwich ELISAs from ELK Biotechnology (Denver, CO, USA): the Cattle CSN1 (Casein Alpha) ELISA Kit, Cattle LTF (lactoferrin) ELISA Kit and Cattle ALP (alkaline phosphatase) ELISA Kit (catalog numbers: ELK8008, ELK10662, ELK1355, respectively). Three proteins, including one enzyme, were selected as markers of correctly performed ELISA tests: CSN1, LTF and ALP. All ELISA tests were dedicated to Serum, Plasma, Tissue homogenates, Cell lysates, Urine, Saliva, Feces, Cell culture supernatants and other biological fluids, Cerebrospinal fluid (CSF) with different beginning steps according to biological material. We used protocol dedicated to tissue homogenates.

According to the manufacturers’ recommendations, the protein fraction should be separated by mechanical homogenization followed by sonication with an ultrasonic cell disruptor. All samples were mechanically homogenized in tubes filled with silica beads by the manufacturer (A&A Biotechnology, Gdynia, Poland). Each sample was divided into four parts, and each part was prepared for analysis using a different protocol. The first part (70 mg of tissue in 700 µl PBS) was mechanically homogenized with PBS solution (MH, baseline).

The second part was mechanically homogenized with PBS solution with the addition of Pierce protease inhibitor tablets (EDTA-free; ThermoFisher Scientific, Waltham, USA) (MH + PI). This allowed for the inhibition of any serine proteases, cysteine proteases, aspartic acid proteases, and aminopeptidases present in the cell lysate samples. The tablets also contain AEBSF, aprotinin, bestatin, E-64, leupeptin, and pepstatin A.

The third part was mechanically homogenized with PBS solution and then sonicated two times with an output power of 60% using a 4710 Series Ultrasonic Homogenizer (Cole-Parmer Instrument, Chicago, IL, USA). Sonication was performed for 5 s. with cooling on ice between cycles (MH + S) for one minute.

The fourth part was mechanically homogenized with PBS and protease inhibitors and sonicated (MH + PI+S) according to protocols described above.

After preparation, the homogenates were centrifuged (5 min at 10,000 × g) and the supernatants collected into clear tubes. For all four parts, all further steps were performed strictly according to the protocols. Briefly, the wells were determined for standards, blanks and samples. Seven standard dilutions were prepared according to the guidelines and added to the appropriate wells (100 µl each). Following this, 100 µl of blank and samples were added to the wells and incubated under foil at 37 °C for 80 min. After incubation, the liquid was discarded from the wells and washed three times with Wash Solution using a Stat Fax^®^ 2600 automatic microplate washer (Awareness Technology, Inc., FL, USA). The plate was dried on absorbent paper and 100 µl of Biotinylated Antibody Working Solution was added to the wells. The plate was incubated at 37 °C for 50 min, then the liquid was discarded from the wells and washed three times. After draining off the remaining liquid, 100 µl of Streptavidin-HRP Working Solution was added to each well and incubated under the same conditions. After incubation, the liquid was discarded from the plate and washed five times. Following this, 90 µl of TMB Substrate Solution was added to each well and the plate was incubated at 37 °C for 20 min in the dark. After incubation, the reaction was stopped by adding 50 µl of Stop Reagent to each well, mixed gently and the measurement was performed. All readings were performed in duplicate for each standard, control and experimental group at 450 nm (Sunrise Microplate Reader, Tecan, Switzerland) and the mean value was taken for analysis.

### Statistical analysis

The effect of parity (lactation number) was examined in the model for preliminary analysis and turned out to be insignificant. Numerical variables (protein concentrations and protein concentration reductions) were examined for normality of distribution using the normal probability Q-Q plots and the Shapiro-Wilk test. As the normality assumption was satisfied, the numerical variables were summarized using arithmetic means with 95% confidence intervals (CI 95%), standard deviation (± SD), and range. Absolute protein concentration reduction was calculated as the difference between the investigated protocol and the baseline MH protocol. The relative reduction in protein concentration was calculated as the ratio of the absolute protein concentration compared to the baseline MH-protocol. The CI 95% for the relative reduction in protein concentration was calculated using Fieller’s method^33^. The data obtained from the samples processed using the four different preparation protocols were compared using the repeated-measure analysis of variance (RM-ANOVA) with the protocols included as paired (related) groups and the udder condition (healthy vs. CoPS-infected) fitted as a fixed effect. The sphericity assumption (homogeneity of variances of between-protocol differences) was verified using Mauchly’s test and was satisfied in the analysis of CSN1 (*p* = 0.498), LTF (*p* = 0.100), and ALP (*p* = 0.099). If significant differences were identified between preparation protocols, they were further analyzed using the Tukey’s *post hoc* test for equal groups. The significance level (α) was set at 0.05. Statistical analysis was performed in TIBCO Statistica 13.3 (TIBCO Software, Inc., Palo Alto, CA, USA), (https://docs.tibco.com/products/tibco-statistica-document-management-system-13-6-0, access: April 2025)

## Supplementary Information

Below is the link to the electronic supplementary material.

Supplementary Material 1

## Data Availability

Data and materials are available upon request from the corresponding author.

## References

[1] Aydin, S. A short history, principles, and types of ELISA, and our laboratory experience with peptide/protein analyses using ELISA. *Peptides* **72**, 4–15 (2015). 25908411 10.1016/j.peptides.2015.04.012

[2] Hornbeck, P. Enzyme-linked immunosorbent assays. *Curr Protoc Immunol Chap. 2*, Unit 2.1 (2001). 10.1002/0471142735.im0201s01 18432761

[3] van Helmond, Z., Heesom, K. & Love, S. Characterisation of two antibodies to oligomeric Aβ and their use in ELISAs on human brain tissue homogenates. *J. Neurosci. Methods*. **176** (2), 206–212 (2009). 18824027 10.1016/j.jneumeth.2008.09.002

[4] Minic, R., Zivkovic, I. & Optimization Validation and Standardization of ELISA. in *Norovirus*IntechOpen, (2020). 10.5772/intechopen.94338

[5] AssayGene Sonication Protocol for Cell Lysis. https://www.assaygenie.com/sonication-protocol-for-cell-lysis?srsltid=AfmBOorlnmUupmrRTSayfW6i1kW2E1JbYr3P6fLCUHNQY_0NWdcJ43xF

[6] Clifton, J. et al. Protease inhibitors as possible pitfalls in proteomic analyses of complex biological samples. *J. Proteom.* **74**, 935–941 (2011). 10.1016/j.jprot.2011.02.010 PMC3107918 21333769

[7] Marathe, K., Patil, R., Vishwakarma, K., Chaudhari, A. & Maheshwari, V. Protease inhibitors and their applications: an overview. *Stud. Nat. Prod. Chem.* **211–242**10.1016/B978-0-444-64185-4.00006-X (2019).

[8] BertinTechnologies. Protein extraction. *Bertin Technologies* https://www.bertin-technologies.com/sample-preparation-homogenizers/application/protein-extraction/

[9] Zhang, G. Protease assays. In *Assay Guidance Manual* (eds Markossian, S. et al.) (Eli Lilly & Company and the National Center for Advancing Translational Sciences, 2012). 22553861

[10] Lamare, M., Taylor, R. G., Farout, L., Briand, Y. & Briand, M. Changes in proteasome activity during postmortem aging of bovine muscle. *Meat Sci.* **61**, 199–204 (2002). 22064010 10.1016/s0309-1740(01)00187-5

[11] Goldberg, A. L. Protein degradation and protection against misfolded or damaged proteins. *Nature* **426**, 895–899 (2003). 14685250 10.1038/nature02263

[12] Rankin, S. A., Christiansen, A., Lee, W., Banavara, D. S. & Lopez-Hernandez, A. Invited review: the application of alkaline phosphatase assays for the validation of milk product pasteurization. *J. Dairy. Sci.* **93**, 5538–5551 (2010). 21094726 10.3168/jds.2010-3400

[13] Ginger, M. R. & Grigor, M. R. Comparative aspects of milk caseins. *Comp. Biochem. Physiol. B Biochem. Mol. Biol.* **124**, 133–145 (1999). 10584297 10.1016/s0305-0491(99)00110-8

[14] Shimazaki, K. & Lactoferrin A marvelous protein in milk. *Nihon Chikusan Gakkaiho*. **71**, 329–347 (2000).

[15] Fang, L. et al. Genome-Wide transcriptional and Post-transcriptional regulation of innate immune and defense responses of bovine mammary gland to Staphylococcus aureus. *Front. Cell. Infect. Microbiol.* **6**, 193 (2016). 28083515 10.3389/fcimb.2016.00193 PMC5183581

[16] Kościuczuk, E. M. et al. Expression patterns of β-defensin and Cathelicidin genes in parenchyma of bovine mammary gland infected with coagulase-positive or coagulase-negative Staphylococci. *BMC Vet. Res.* **10**, 246 (2014). 25286984 10.1186/s12917-014-0246-z PMC4194403

[17] Sule, R., Rivera, G. & Gomes, A. V. Western blotting (immunoblotting): history, theory, uses, protocol and problems. *Biotechniques* **75**, 99–114 (2023). 36971113 10.2144/btn-2022-0034 PMC12303220

[18] Full Moon BioSystems Inc. Antibody Array User’s Guide.

[19] Im, H. & Snyder, M. Preparation of Recombinant protein spotted arrays for proteome-wide identification of kinase targets. *Curr Protoc. Protein Sci. Chap* **27**, (2013). Unit 27.4. 10.1002/0471140864.ps2704s72 PMC4414062 23546622

[20] Pchelintsev, N. A., Adams, P. D. & Nelson, D. M. Critical parameters for efficient sonication and improved chromatin Immunoprecipitation of high molecular weight proteins. *PLoS One*. **11**, e0148023 (2016). 26821228 10.1371/journal.pone.0148023 PMC4731078

[21] Özbek, B. & Ulgen, K. The stability of enzymes after sonication. *Process Biochem.* **35**, 1037–1043 (2000).

[22] Liu, D., Zeng, X. A., Sun, D. W. & Han, Z. Disruption and protein release by ultrasonication of yeast cells. *Innovative Food Sci. Emerg. Technol.* **18**, 132–137 (2013).

[23] Mishra, M., Tiwari, S. & Gomes, A. V. Protein purification and analysis: next generation Western blotting techniques. *Expert Rev. Proteom.* **14**, 1037–1053 (2017). 10.1080/14789450.2017.1388167 PMC6810642 28974114

[24] Lee, J., Chan, S. L. & Mattson, M. P. Adverse effect of a presenilin-1 mutation in microglia results in enhanced nitric oxide and inflammatory cytokine responses to immune challenge in the brain. *Neuromolecular Med.* **2**, 29–45 (2002). 12230303 10.1385/NMM:2:1:29

[25] Zhang, P., Nelson, S., Holmes, M. C., Summer, W. R. & Bagby, G. J. Compartmentalization of macrophage inflammatory Protein-2, but not Cytokine-Induced neutrophil Chemoattractant, in rats challenged with intratracheal endotoxin. *Shock* **17**, 104 (2002). 11837784 10.1097/00024382-200202000-00004

[26] Phelan, H., Stahls, P., Hunt, J., Bagby, G. J. & Molina, P. E. Impact of alcohol intoxication on hemodynamic, metabolic, and cytokine responses to hemorrhagic shock. *J. Trauma.* **52**, 675–682 (2002). 11956381 10.1097/00005373-200204000-00010

[27] Fernandez-Botran, R. et al. Targeting of glycosaminoglycan-cytokine interactions as a novel therapeutic approach in allotransplantation. *Transplantation* **74**, 623–629 (2002). 12352877 10.1097/00007890-200209150-00007

[28] Ungvarski, P. J. & Rottner, J. E. Errors in prescribing HIV-1 protease inhibitors. *J. Assoc. Nurses AIDS Care*. **8**, 55–61 (1997). 9260151 10.1016/S1055-3290(97)80013-1

[29] Johansson, M., Åkerstedt, M., Li, S. & Zamaratskaia, G. Sternesjö Lundh, Å. Casein breakdown in bovine milk by a field strain of Staphylococcus aureus. *J. Food Prot.* **76**, 1638–1642 (2013). 23992512 10.4315/0362-028X.JFP-13-112

[30] Zalewska, M. et al. The quality and technological parameters of milk obtained from dairy cows with subclinical mastitis. *J. Dairy. Sci.* **108**, 1285–1300 (2025). 39521420 10.3168/jds.2024-25346

[31] Babaei, H., Mansouri-Najand, L., Molaei, M. M., Kheradmand, A. & Sharifan, M. Assessment of lactate dehydrogenase, alkaline phosphatase and aspartate aminotransferase activities in cow’s milk as an indicator of subclinical mastitis. *Vet. Res. Commun.* **31**, 419–425 (2007). 17268916 10.1007/s11259-007-3539-x

[32] Korwin-Kossakowska, A. et al. Gene expression adjustment of inflammatory mechanisms in dairy cow mammary gland parenchyma during host defense against Staphylococci. *Annals Anim. Sci.* **22**, 903–913 (2022).

[33] Fieller, E. C. Some problems in interval Estimation. *J. Royal Stat. Soc. Ser. B (Methodological)*. **16**, 175–185 (1954).

