# Supplementary material for: Effect of sonication and protease inhibitors on Elisa quantification of selected proteins in bovine udder tissue homogenates
Source: Sci Rep. 2026 Feb 5;16:7366. doi: 10.1038/s41598-026-38653-x (PMC12923574; doi:10.1038/s41598-026-38653-x)
Supplement: Supplementary file 1 — Supplementary Material 1 [file 41598_2026_38653_MOESM1_ESM.docx]

Supplementary file

Table S1. Overall protein concentration [ng/ml] in the udder samples following sample preparation using four different protocols, presented as the arithmetic mean, standard deviation (±SD) and range in parentheses. MH – Mechanical Homogenization, MH+PI – Mechanical Homogenization + Protease Inhibitors, MH+S – Mechanical Homogenization + Sonication, MH+PI+S – Mechanical Homogenization + Protease Inhibitors + Sonication

|  | Sample preparation protocol | | | |  |  |
| --- | --- | --- | --- | --- | --- | --- |
| Protein | MH | MH+PI | MH+S | MH+PI+S | F-statistic (df1=3, df2=60) | p-value |
| CSN1 | 11.58 ± 5.52 (2.97 – 18.15) | 9.69 ± 4.62 (1.53 – 16.48) | 9.16 ± 5.02 (1.44 – 17.20) | 7.73 ± 4.98 (0.57 – 16.21) | 10.5 | <0.001 |
| LTF | 62.67 ± 28.70 (20.08 – 125.5) | 40.60 ± 17.44 (12.85 – 88.78) | 32.77 ± 18.95 (13.99 – 76.17) | 26.65 ± 14.64 (7.94 – 59.90) | 39.6 | <0.001 |
| ALP | 6.98 ± 3.05 (3.04 – 13.74) | 5.33 ± 1.96 (1.43 – 9.87) | 3.70 ± 1.90 (1.53 – 7.65) | 2.81 ± 1.26 (0.97 – 5.81) | 68.1 | <0.001 |

Table S2. Protein concentration [ng/ml] in the udder samples from coagulase-positive staphylococcus (CoPS)-infected and healthy cows following sample preparation using four different protocols presented as the arithmetic mean, standard deviation (±SD) and range in parentheses. MH – Mechanical Homogenization, MH+PI – Mechanical Homogenization + Protease Inhibitors, MH+S – Mechanical Homogenization + Sonication, MH+PI+S – Mechanical Homogenization + Protease Inhibitors + Sonication

|  |  | Sample preparation protocol | | | |
| --- | --- | --- | --- | --- | --- |
| Protein | Group | MH | MH+PI | MH+S | MH+PI+S |
| CSN1 | CoPS-infected | 14.27 ± 4.02 (6.09 – 17.7) | 12.00 ± 3.64 (5.82 – 16.48) | 11.68 ± 3.97 (3.99 – 17.20) | 10.30 ± 3.58 (5.42 – 16.21) |
|  | Healthy | 8.88 ± 5.65 (2.97 – 18.15) | 7.38 ± 4.45 (1.53 – 14.6) | 6.64 ± 4.81 (1.44 – 14.42) | 5.17 ± 4.98 (0.57 – 15.15) |
|  | F-statistic (df1=1, df2=20) | 8.92 |  |  |  |
|  | p-value | 0.007 |  |  |  |
| LTF | CoPS-infected | 72.80 ± 25.74 (38.40 – 125.5) | 47.36 ± 19.84 (15.24 – 88.78) | 37.43 ± 19.03 (14.35 – 72.57) | 31.28 ± 13.88 (9.56 – 59.9) |
|  | Healthy | 52.54 ± 29.00 (20.08 – 108.5) | 33.85 ± 12.04 (12.85 – 55.71) | 28.12 ± 18.56 (13.99 – 76.17) | 22.02 ± 14.49 (7.94 – 55.02) |
|  | F-statistic (df1=1, df2=20) | 3.24 |  |  |  |
|  | p-value | 0.087 |  |  |  |
| ALP | CoPS-infected | 8.17 ± 2.78 (3.97 – 13.74) | 5.87 ± 1.98 (2.75 – 9.87) | 4.28 ± 1.97 (2.14 – 7.65) | 3.24 ± 1.21 (1.52 – 5.5) |
|  | Healthy | 5.78 ± 2.95 (3.04 – 12.64) | 4.78 ± 1.87 (1.43 – 7.79) | 3.11 ± 1.71 (1.53 – 7.21) | 2.39 ± 1.21 (0.97 – 5.81) |
|  | F-statistic (df1=1, df2=20) | 3.08 |  |  |  |
|  | p-value | 0.095 |  |  |  |

c

b

a

Fig. S1. Concentration of a) casein alpha (CSN1), b) lactoferrin (LTF), and c) alkaline phosphatase (ALP) in the udder samples from coagulase-positive staphylococcus (CoPS)-infected and healthy cows prepared using 4 different protocols presented as the arithmetic means with 95% confidence intervals. MH – Mechanical Homogenization, MH+PI – Mechanical Homogenization + Protease Inhibitors, MH+S – Mechanical Homogenization + Sonication, MH+PI+S – Mechanical Homogenization + Protease Inhibitors + Sonication

c

b

a

Fig. S2. Mean (CI 95%) reduction of a) casein alpha (CSN1), b) lactoferrin (LTF), and c) alkaline phosphatase (ALP) concentration following sample preparation using three different protocols: mechanical homogenization combined with protease inhibition (MH+PI), mechanical homogenization followed by sonication (MH+S), and mechanical homogenization combined with protease inhibition and sonication (MH+PI+S) with mechanical homogenization (MH) considered as a baseline protocol.
